# Characterisation of a solvent-tolerant haloarchaeal (*R*)-selective transaminase isolated from a Triassic period salt mine

**DOI:** 10.1007/s00253-019-09806-y

**Published:** 2019-05-23

**Authors:** Stephen A. Kelly, Damian J. Magill, Julianne Megaw, Timofey Skvortsov, Thorsten Allers, John W. McGrath, Christopher C. R. Allen, Thomas S. Moody, Brendan F. Gilmore

**Affiliations:** 1 School of Pharmacy, Queen’s University Belfast, Belfast, UK; 2 School of Biological Sciences, Queen’s University Belfast, Belfast, UK; 3 School of Life Sciences, University of Nottingham, Queen’s Medical Centre, Nottingham, UK; 4 Almac, Department of Biocatalysis & Isotope Chemistry, 20 Seagoe Industrial Estate, Craigavon, UK; 5 Arran Chemical Company Limited, Unit 1 Monksland Industrial Estate, Athlone, Co. Roscommon Ireland

**Keywords:** Archaea, Biocatalysis, Halophile, Organic solvent, Transaminase

## Abstract

Transaminase enzymes (TAms) are becoming increasingly valuable in the chemist’s toolbox as a biocatalytic route to chiral amines. Despite high profile successes, the lack of (*R*)-selective TAms and robustness under harsh industrial conditions continue to prove problematic. Herein, we report the isolation of the first haloarchaeal TAm (BC61-TAm) to be characterised for the purposes of pharmaceutical biocatalysis. BC61-TAm is an (*R*)-selective enzyme, cloned from an extremely halophilic archaeon, isolated from a Triassic period salt mine. Produced using a *Haloferax volcanii*–based expression model, the resulting protein displays a classic halophilic activity profile, as well as thermotolerance (optimum 50 °C) and organic solvent tolerance. Molecular modelling predicts the putative active site residues of haloarchaeal TAms, with molecular dynamics simulations providing insights on the basis of BC61-TAm’s organic solvent tolerance. These results represent an exciting advance in the study of transaminases from extremophiles, providing a possible scaffold for future discovery of biocatalytic enzymes with robust properties.

**Electronic supplementary material:**

The online version of this article (10.1007/s00253-019-09806-y) contains supplementary material, which is available to authorized users.

## Introduction

Chiral amines are valuable building blocks for the pharmaceutical industry, representing versatile intermediates in the synthesis of active pharmaceutical ingredients (APIs). Their synthesis using conventional chemical means can suffer from a number of critical drawbacks, including the need for volatile organic solvents and toxic transition metal catalysts, as well as a lack of enantioselectivity in a single step (Malik et al. 2012).

Biocatalytic routes to optically active amines have emerged as a ‘green’ alternative to conventional synthetic chemistry approaches, expanding the chemist’s toolbox and affording an economically viable approach for the production of these valuable compounds. The ability of biocatalysts to operate in aqueous media, as well as at ambient temperature and neutral pH, makes them extremely desirable and useful in streamlining API synthesis.

To date, a number of enzymes have been employed in the production of chiral amines, including hydrolases, lyases, oxidases and dehydrogenases (Busto et al. 2011; Kroutil et al. 2013; Sharma et al. 2017). The demand for efficient production of optically active amines has also driven research into the use of transaminase enzymes (TAms) for this purpose.

TAms are pyridoxal phosphate (PLP)–dependent enzymes capable of transferring an amine group from a donor molecule to a prochiral ketone, resulting in the formation of a chiral amine. Their use in industrial biocatalysis has been the subject of a number of recent reviews (Malik et al. 2012; Ghislieri and Turner 2013; Kohls et al. 2014; Guo and Berglund 2017; Kelly et al. 2018). TAms have been employed in the synthesis of a number of drug molecules (Fuchs et al. 2010; Mangion et al. 2012; Midelfort et al. 2013), with their capabilities best showcased in the production of the antidiabetic drug sitagliptin (Savile et al. 2010). In this highly successful example, a bacterial TAm, optimised by protein engineering, brought about the conversion of the prochiral ketone prositagliptin to the chiral amine molecule sitagliptin. As well as replacing a number of chemical steps, including a hydrogenation step requiring expensive high-pressure equipment, this biocatalytic approach also improved overall yield and reduced waste.

TAms, as with other enzymes, are not without drawbacks. A narrow substrate scope and an inability to deal with challenging conditions often demanded by industrial processes, highlight the need for improvement of the current TAm toolbox. Whilst TAms possess the advantage of being highly enantioselective, a consideration which is critical for pharmaceutical production, the lack of (*R*)-selective TAms in the current catalogue is an issue which must be addressed. Important steps have been taken in order to overcome this barrier, including the use of rational design to reverse the enantiopreference of TAms (Svedendahl et al. 2010; Humble et al. 2012), but the issue remains problematic. The discovery of naturally (*R*)-selective TAms, whilst challenging, is the most obvious route to address this current impasse.

Shortfalls encountered with currently available TAms have led to a number of approaches for improvement and process-specific optimisation. Protein engineering has brought about significant advances in both activity and substrate range (Savile et al. 2010; Nobili et al. 2015; Dourado et al. 2016), whilst another viable option is the search for novel enzymes with naturally optimised capabilities, such as enzymes from environments whose conditions more closely mimic those of industrial processes. With the majority of current enzymes derived from culturable mesophilic bacteria, the quest for new TAms has been expanded to include metagenomic mining, including those from the archaeal domain, and from extreme environments (Littlechild 2015; Baud et al. 2017; Bezsudnova et al. 2017).

Interrogation of extreme environments has focused heavily on enzymes from thermophilic organisms, which have been studied in much greater detail relative to their halophilic counterparts. Scarcely is this trend more evident than with halophilic TAms, where the number of enzymes has been quite limited to date (Muriana et al. 1991). This is surprising given the range of extreme conditions halophilic microorganisms, and often by extension their enzymes, can tolerate not only functioning at high salt concentrations but also with increased temperatures, in organic solvent, and often at elevated pH (Hough and Danson 1999; Doukyu and Ogino 2010; Amoozegar et al. 2017). This ‘polyextremophilicity’ indicates that halophilic enzymes have the ability to tolerate a range of harsh conditions, and with it the ability to effectively ‘green’ a multitude of industrial processes.

The most extreme adaptations of halophiles are generally the reserve of haloarchaea. Archaea represents the third domain of life, proposed for the first time as recently as 1990 (Woese et al. 1990). As such, they remain hugely understudied relative to their bacterial counterparts, and represent a potentially vast untapped resource of novel and diverse biocatalysts.

In this study, we describe a TAm from a haloarchaeon, *Halorubrum* sp. CSM-61, isolated from a Triassic period halite deposit (formed circa 220–250 mya). To our knowledge, this is the first example of a haloarchaeal TAm to be cloned and overexpressed and investigated for its potential use in biocatalysis.

## Materials and methods

### Organism isolation and identification

Organism CSM-61 was isolated by inoculating media containing (per litre) rock salt from Kilroot salt mine (250 g), yeast extract (10 g), casein hydrolysate (7.5 g) and glycerol (10 mL) with 100 μL undiluted brine collected from a brine stream within the mine. Identification was based on the 16S rRNA gene and performed using colony PCR as described previously (Supporting information; Kelly et al. 2017), using the universal primers ARCH21F (5′-TTCCGGTTGATCCYGCCGGA-3′) and 958R (5′-YCCGGCGTTGAMTCCAATT-3′).

### DNA extraction and PCR amplification of BC61-TAm gene

Genomic DNA was extracted and sequenced, and the assembled genome annotated and BC61-TAm identified as described previously (Supporting information; Kelly et al. 2017). Forward (5′-TTTTTT**CCATGG**GATTCGACGAGATGGAC-3′) and reverse (5′-TTTTTT**GAATTC**TTATACGTAGGTGAACCAGTCGTC-3′) primers were designed specific to the gene sequence (Invitrogen, UK) with restriction sites included for *Nco*I and *Eco*RI-HF respectively (highlighted in bold). The BC61-TAm gene was amplified and checked as described previously (Supporting information; Kelly et al. 2017).

### Preparation of pTA1228/BC61-TAm plasmid and transformation of *Escherichia coli*

The pTA1228/BC61-TAm plasmid was prepared as follows: 25 μL pTA1228 vector was cut using restriction enzymes *Pci*I (1 μL) in Tango buffer (3 μL) (Thermo Fisher Scientific, UK) and *Eco*RI-HF (1 μL) in CutSmart buffer (3 μL) in two separate single digests, with purification between each digest using a GeneJet PCR Purification kit (Thermo Fisher Scientific, UK). Alkaline Calf Intestinal Phosphatase (CIP) (NEB) was subsequently added to the pTA1228 mix and incubated for a further 1 h at 37 °C. BC61-TAm gene was cut using *Nco*I (1 μL) and *Eco*RI-HF (1 μL) in CutSmart buffer (3 μL) in a double digest, followed by PCR purification. Ligation and transformation of One Shot® TOP10 Chemically Competent *E. coli* cells were carried out as described previously (Supporting information; Kelly et al. 2017).

### Transformation of *Hfx. volcanii* H1424 with pTA1228/BC61-TAm

*Hfx. volcanii* H1424 cells (Stroud et al. 2012) were transformed using a protocol described previously by the Allers group (Nuttall et al. 2009). A primary culture of *Hfx. volcanii* H1424 (10 mL, OD_550_ ≈ 0.8) was pelleted at 6000 rpm for 8 min. The pellet was suspended in 2 mL 50 mM Tris-HCl, pH 8.5 buffer containing NaCl (1 M), KCl (27 mM) and 15% sucrose, before pelleting again with the same conditions. The pellet was gently resuspended in 600 μL unbuffered spheroplasting solution containing NaCl (1 M), KCl (27 mM) and 15% sucrose, and 200 μL transferred to a clean, 2-mL round-bottomed tube for each transformation. 0.5 M EDTA, pH 8.0 (20 μL), was added to the side of the tube, inverted to mix and left for 10 min at room temperature. DNA samples were prepared with 10 μL pTA228/BC61-TAm plasmid (~ 1 μg), unbuffered spheroplasting solution (15 μL) and 0.5 M EDTA, pH 8.0 (5 μL). This mixture was added to the cell suspension as described before and left for 5 min at room temperature. An equal volume of 60% PEG_600_ in unbuffered spheroplasting solution was added in the same manner as before, the tube shaken horizontally to mix and left for 30 min at room temperature. The mixture was diluted with 1.5 mL spheroplast dilution solution (Nuttall et al. 2009), inverted to mix and left for 2 min at room temperature, before pelleting at 6000 rpm for 8 min. The supernatant was discarded and 1 mL regeneration solution (Nuttall et al. 2009) was added, with a wide-bore pipette used to transfer the pellet and solution to a 5-mL sterile tube. This was incubated undisturbed at 45 °C for 2 h, before leaving for a further 4 h with rotation. Following incubation, cells were transferred to a 2-mL round-bottomed tube and centrifuged at 6000 rpm for 8 min, with the resultant pellet resuspended in 1 mL transformant dilution solution (Nuttall et al. 2009). Suspensions were diluted further to 10^−1^ and 10^−2^ with transformant dilution solution and plated on Hv-Ca+ agar (Nuttall et al. 2009) and incubated at 45 °C. After around 1 week, colonies were restreaked on Hv-Ca+ agar for purity, with fresh colonies used to inoculate 5 mL Hv-Ca+ broth and incubated at 45 °C with rotation. Protein expression was carried out based on an adapted method described previously (Liliensiek et al. 2013). When primary cultures reached OD_550_ ≈ 1.0, they were used to inoculate 1 L Hv-YPC broth (Allers et al. 2004) containing 5 mM l-tryptophan and incubated at 45 °C with rotation to an OD_550_ ≈ 1.0. Following incubation, the culture was pelleted via centrifugation, freeze-thawed at − 80 °C, suspended in 50 mM Tris-HCl buffer, pH 7.5 containing 2 M NaCl and 2 mM EDTA, and sonicated on ice. This was further centrifuged to remove cell debris. The supernatant was clarified using 0.8 μM filters and purified as described below.

### His-tag-mediated purification and expression analysis of BC61-TAm protein

BC61-TAm protein was purified from clarified cell-free extract (CFE) by immobilised metal affinity chromatography (IMAC) using the N-terminal 6× His-tag encoded by the pTA1228 vector. An ÄKTA Prime Plus Liquid Chromatography System was used to load the CFE on a HisTrap™ HP 1 mL column (both GE Healthcare Life Sciences, UK) at a rate of 1 mL min^−1^. This was washed using buffer containing 20 mM HEPES, pH 7.5, 2 M NaCl, 20 mM imidazole, 1 mM PMSF made up to 1 L with dH_2_O. Purified protein was eluted in 1 mL fractions using buffer containing 20 mM HEPES, pH 7.5, 2 M NaCl, 500 mM imidazole, 1 mM PMSF adjusted to 1 L with dH_2_O. Recombinant BC61-TAm protein was analysed via SDS-PAGE.

### HPLC-based screening assay employing (*R*)-methylbenzylamine as amino donor

(*R*)-Methylbenzylamine (MBA) (25 mM) and PLP (0.5 mM in potassium phosphate buffer, 100 mM, pH 8.0, 2 M KCl), and α-ketoglutarate (α-KG) (5 mM) were added to a 96-well deep well plate to give a total volume of 180 μL. The reaction was initiated by the addition of 20 μL purified BC61-TAm to a final working concentration of 1 mg/mL. After incubation for 16 h at 45 °C and 1200 rpm, the reaction was quenched with 800 μL 62.5% acetonitrile and denatured protein was removed by centrifugation. Two hundred microlitres of supernatant was removed for analysis by HPLC using an XSelect® CSH™ C18 5 μM column (4.6 × 250 mm) and an Agilent 1260 Infinity model. Detection of acetophenone coproduct was carried out at 240 nm, in an adaptation of a previously described study (Schätzle et al. 2009). The concentration of acetophenone coproduct formation was determined over 11 min using a gradient of 5–95% organic solvent (where A = dH_2_O with 0.1% H_3_PO_4_ and B = acetonitrile with 0.1% H_3_PO_4_). Acetophenone eluted at a retention time of 8.6 min. Assays using both BC61-TAm and elution buffer negative controls were carried out in triplicate for each substrate, with an average conversion taken for each and values for negative control subtracted from BC61-TAm values.

To assess the effect of temperature on enzyme performance, assays were set up as described above but incubated at different temperatures, ranging from 30 to 60 °C.

To assess the effect of changing pH on enzyme performance, assays were set up using a universal buffer (Davies 1959), containing 25 mM citric acid, 25 mM KH_2_PO_4_, 25 mM Tris, 12.5 mM Na_2_B_4_O_7_ and 2 M KCl, adjusted using NaOH, with pH values from pH 6.0 to 11.0 investigated.

To assess the effect of organic solvents on enzyme performance, more concentrated stock solutions of potassium phosphate buffer (pH 8.0) were prepared to reduce the aqueous volume in the reaction, with PLP concentrations also adjusted in buffer solutions accordingly. Once diluted by the required amount of organic solvent, the overall concentrations of potassium phosphate buffer (pH 8.0), PLP and KC1 in solution remained at 100 mM, 0.5 mM and 2 M as before. Concentrations of organic solvents investigated ranged from 0 to 30%.

Kinetic parameters were determined using the reaction described above, with α-KG concentration varied from 1 to 20 mM and amino donor concentration maintained at 5 equivalents for each reaction. KCl and DMF concentrations were 1 M and 30% respectively, with each reaction carried out in triplicate. Reactions were quenched with 800 μL 62.5% acetonitrile at regular time intervals and denatured protein was removed by centrifugation. The concentration of acetophenone coproduct formation was determined as described above. BC61-TAm concentration was determined using a combination of a Qubit® 2.0 Fluorometer (Invitrogen, UK) and SDS-PAGE densitometry.

### Molecular modelling and substrate docking

Molecular models of BC61-TAm were constructed using the I-TASSER server (https://zhanglab.ccmb.med.umich.edu/I-TASSER) followed by energy minimisation using Yasara (Krieger et al. 2009). Molecular models of PMP and α-KG were constructed in Ascalaph Designer and subject to 1000 steps of steepest descent energy minimisation. The fidelity of the BC61-TAm model was assessed using the tools of the Whatif server along with Ramachandran analysis. Molecular models and docked complexes were viewed and manipulated in PyMol (DeLano 2002).

AutoDock Vina was utilised for the docking of both PMP and α-KG in two separate docking events (Trott and Olson 2010). Yasara energy minimisation was conducted following each docking step resulting in the production of the final docked complex. Construction of residue interaction maps was carried out using LigPlus followed by visualisation in PyMol.

### Molecular dynamics simulations

Hydrogen atoms were added to simulated systems according to protonation states of individual residues with protonatable side chains at physiological pH following pKa analysis using PROPKA (Rostkowski et al. 2011). A molecule of DMF was constructed within Ascalaph Designer and subject to 1000 steps of steepest descent energy minimisation. Parameterisation of DMF was carried out using the PRODG server and a compatible GROMOS topology subsequently produced (Schüttelkopf and Van Aalten 2004).

All proteins were immersed within truncated octahedral boxes of explicit solvent (TIP3P water) with a minimum clearance of 20 Å between periodic images for starting configurations. Solvent molecules were replaced with 2433 molecules of DMF to leave a final concentration of 30% in DMF simulations. The GROMOS96 54a7 force field was utilised for all simulations. Solvent molecules were replaced with Na^+^ and Cl^−^ ions to neutralise all charges and leave a final physiological concentration of 100 mM by using the genion facility of Gromacs v4.6.5 (Hess et al. 2008).

Steepest descent energy minimisation was carried out on all simulations with an energy step of 0.01, until a maximum potential force of < 1000 kJ Mol^−1^ was achieved. The Verlet cutoff scheme was utilised with particle mesh Ewald (PME) treatment of electrostatic interactions (Grubmüller et al. 1991; Darden et al. 1993). Energy, pressure and temperature were monitored for the entirety of the simulation set up. NVT equilibration was carried out for 100 ps with a 2-fs integration step. The LINCS algorithm with holonomic constraints was used to constrain all bonds (Hess et al. 1997). PME was implemented for long-range electrostatics with a 9 Å cutoff for the real-space term and non-bonded Van der Waals interactions were calculated using Lennard-Jones 12-6 potentials with a 9 Å cutoff. Velocities were derived from a Maxwell distribution. A similar set up was used for a subsequent NPT equilibration with Parrinello-Rahman pressure coupling implemented (Parrinello and Rahman 1980). Periodic boundary conditions were used for the systems with cutoff radii of 1 nm. Systems then underwent simulation for a total time of 10 ns with 2-fs integration time. Coordinates were saved every 10 ps. Triplicate simulations were conducted for both BC61-TAm in water and in 30% DMF and were run on the high performance computing cluster at Queen’s University Belfast.

Analysis of system properties was conducted using intrinsic tools of Gromacs v4.6.5 along with Chimera and PyMol for the manipulation of models and their subsequent visualisation. Graphs were subsequently produced within the R programming language.

### BC61-TAm gene and protein sequences

BC61-TAm sequence data was deposited using the BankIt sequence submission tool. Gene sequence data can be found in the GenBank database with Accession Number MK248484.1 and protein sequence data can be found in the GenPept database with Accession Number AZS32940.1.

## Results

### Organism identification and gene selection

The organism, from which the TAm enzyme was cloned and expressed, was isolated from a brine sample from Kilroot salt mine and given the designation CSM-61. Using the BLASTn function of the NCBI database, the organism was found to be a member of the genus *Halorubrum*, with the closest neighbour deemed to be *Halorubrum saccharovorum* JCM 8865.

On uploading the assembled contigs from Whole Genome Sequencing data to the RAST server (Aziz et al. 2008), the subsystems search tool revealed 19 results using the search term ‘aminotransferase’. The gene chosen was 930 bp in length and the corresponding protein was given the designation BC61-TAm.

### Expression and phylogenetic analysis

**Fig. 1:**
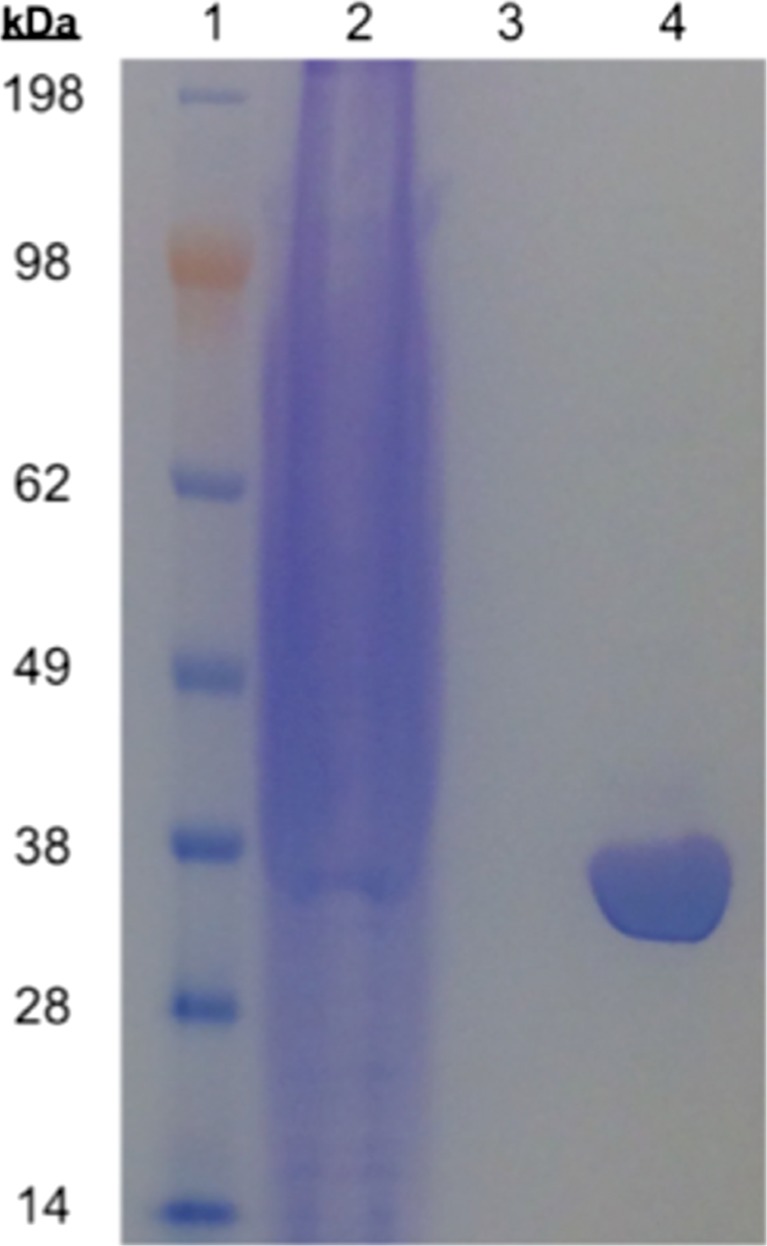
SDS-PAGE of pre-purified clarified cell-free extract (lane 2) and BC61-TAm fraction, purified by IMAC (lane 4), alongside SeeBlue® Plus2 Protein standard (lane 1) and buffer used for protein elution (lane 3)

SDS-PAGE analysis following IMAC purification showed a 34–35-kDa protein, commensurate with gene length and predicted size from the amino acid sequence (Fig. 1). Using a BLASTp search of the NCBI database, the closest neighbour to BC61-TAm was deemed to be a BCAT (fold type IV of the PLP superfamily), from *Halorubrum* sp. (Hp-TAm), at 94% identity (NCBI Reference Sequence WP_099256159.1).

**Fig. 2:**
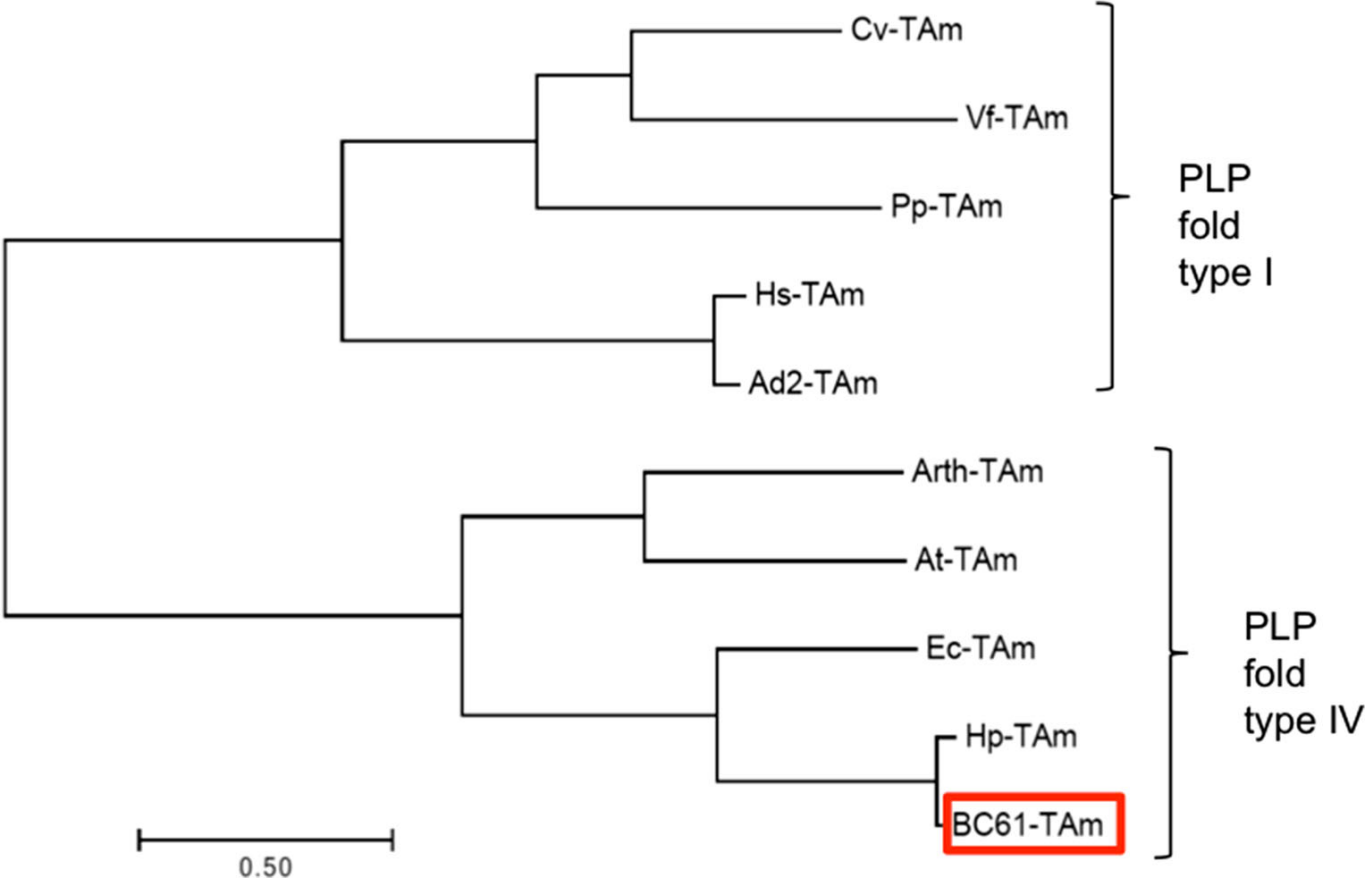
Multiple alignment of amino acid sequences of BC61-TAm with a number of previously reported TAm sequences, including (S)-selective TAms from mesophilic bacteria (Cv-TAm, Vf-TAm, and Pp-TAm) and halophilic bacteria (Hs-TAm and Ad2-TAm), (*R*)-selective ω-TAms from bacteria and fungi (Arth-TAm and At-TAm respectively), and BCATs from bacteria (Ec-TAm) and haloarchaea (Hp-TAm and BC61-TAm)

The phylogeny of BC61-TAm was investigated alongside a number of previously described TAms. Sequences were selected from both PLP fold types I and IV for comparison. PLP fold type I enzymes included (*S*)-selective ω-TAms from mesophilic bacteria (Vf-TAm from *Vibrio fluvialis* (PDB: 3NUI_A), Cv-TAm from *Chromobacterium violaceum* (NCBI Reference Sequence: WP_011135573.1), Pp-TAm from *Pseudomonas putida* (UniProtKB/Swiss-Prot: P28269.1)) and halophilic bacteria (Hs-TAm from *Halomonas* sp. (GenBank: KUJ87738.1) and Ad2-TAm (Kelly et al. 2017)). PLP fold type IV enzymes included (*R*)-selective ω-TAms (Arth-TAm from *Arthrobacter* sp. KNK168 (Genbank: BAK39753.1) and At-TAm from *Aspergillus terreus* (PDB: 4CE5_B)) and BCATs from both mesophilic bacteria (Ec-TAm from *E. coli* (PDB: 1IYE_A)) and haloarchaea (Hp-TAm) (Fig. 2).

BC61-TAm contained a higher proportion of the acidic amino acids, aspartate and glutamate (19.42%), versus those from the mesophilic bacterial TAms, Cv-TAm, Pp-TAm, Vf-TAm, Ec-TAm, and Arth-TAm (12.21%). The overall proportion of lysine residues was lower in BC61-TAm at 1.94% than with the same mesophilic bacterial TAms at 3.96%.

### Evaluation of enzyme performance under varying reaction parameters

**Fig. 3:**
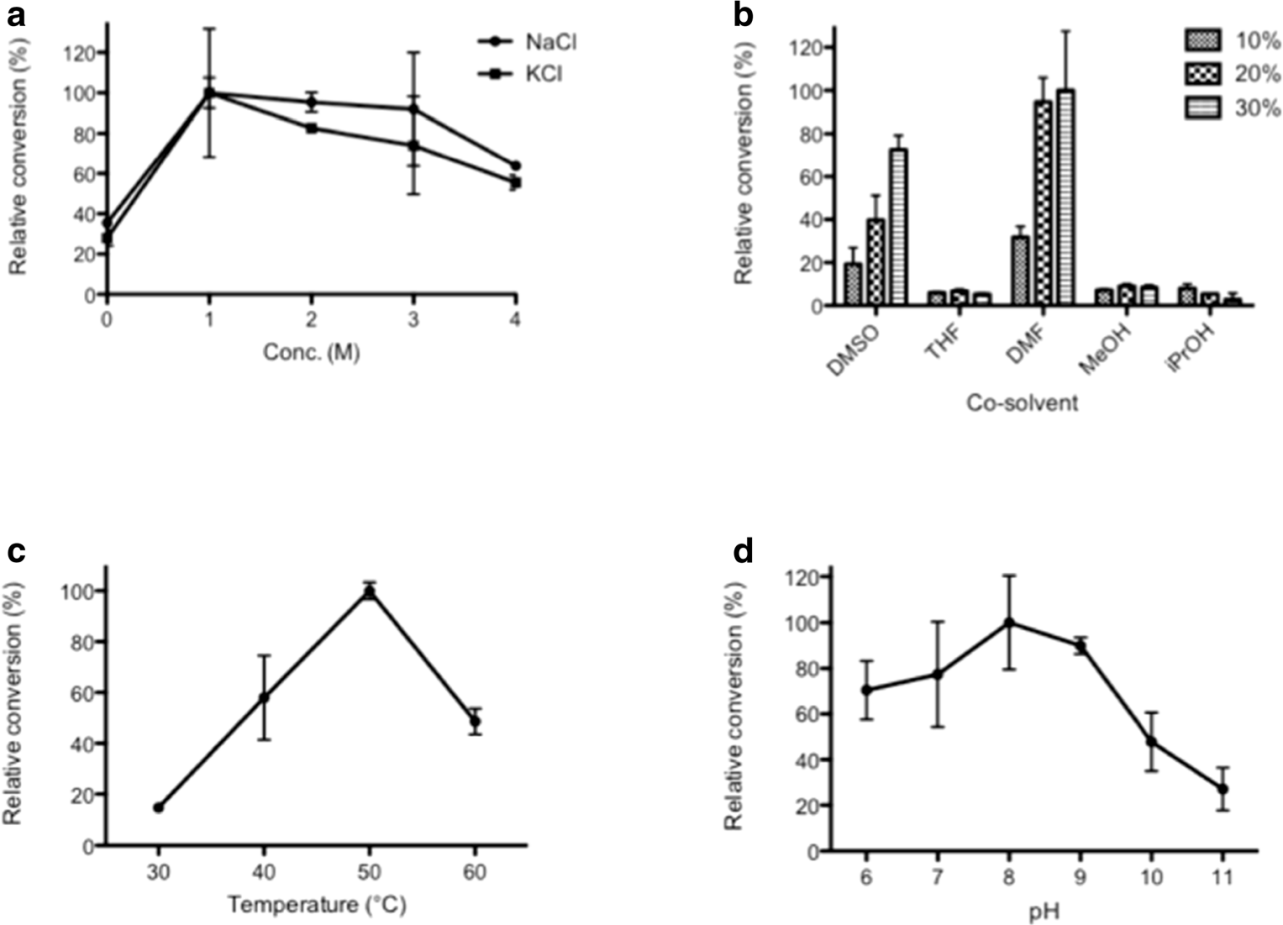
The effect of changing various parameters on the amination of α-KG using purified BC61-TAm and (*R*)-MBA as amino donor, with acetophenone formation measured at 240 nm. Plotted values are the mean of triplicate measurements, with error bars representing ± standard deviation. In each graph, conversion is reported relative to the highest value observed for each parameter. (Abbreviations shown in panel B: DMSO, dimethyl sulfoxide; THF, tetrahydrofuran; DMF, dimethylformamide; MeOH, methanol; iPrOH, isopropanol)

BC61-TAm was shown to be an (*R*)-selective TAm with its ability to utilise (*R*)-MBA as an amino donor, whilst not accepting the (*S*)-isomer. For both NaCl and KCl, conversion of α-KG improved with the addition of salt to the reaction versus no salt, with 100% relative conversion seen at 1 M for both salts. Conversions remained high with increasing salinities beyond 1 M, with 95.4, 91.9 and 63.8% relative conversions observed for 2, 3 and 4 M NaCl respectively, relative to the conversion seen at 1 M (Fig. 3a).

BC61-TAm performed well in a number of scenarios with increasing levels of organic solvent (Fig. 3b). Greatest overall conversions were seen with 30% DMF in the reaction mix, although BC61-TAm also performed well when the reaction was supplemented with DMSO.

Optimal conversion in the BC61-TAm-catalysed reaction was observed at 50 °C (Fig. 3c) and pH 8.0 (Fig. 3d).

Despite being annotated as a BCAT, BC61-TAm was able to produce 17.4% conversion of α-KG using (*R*)-MBA as amino donor, with a specific activity of 73.22 U/mg, where one unit is defined as the amount of enzyme producing 1 nmol of acetophenone per minute. Km was determined as 3.37 mM and kcat as 2.52 min^−1^.

### Modelling and molecular dynamics of BC61-TAm

**Fig. 4:**
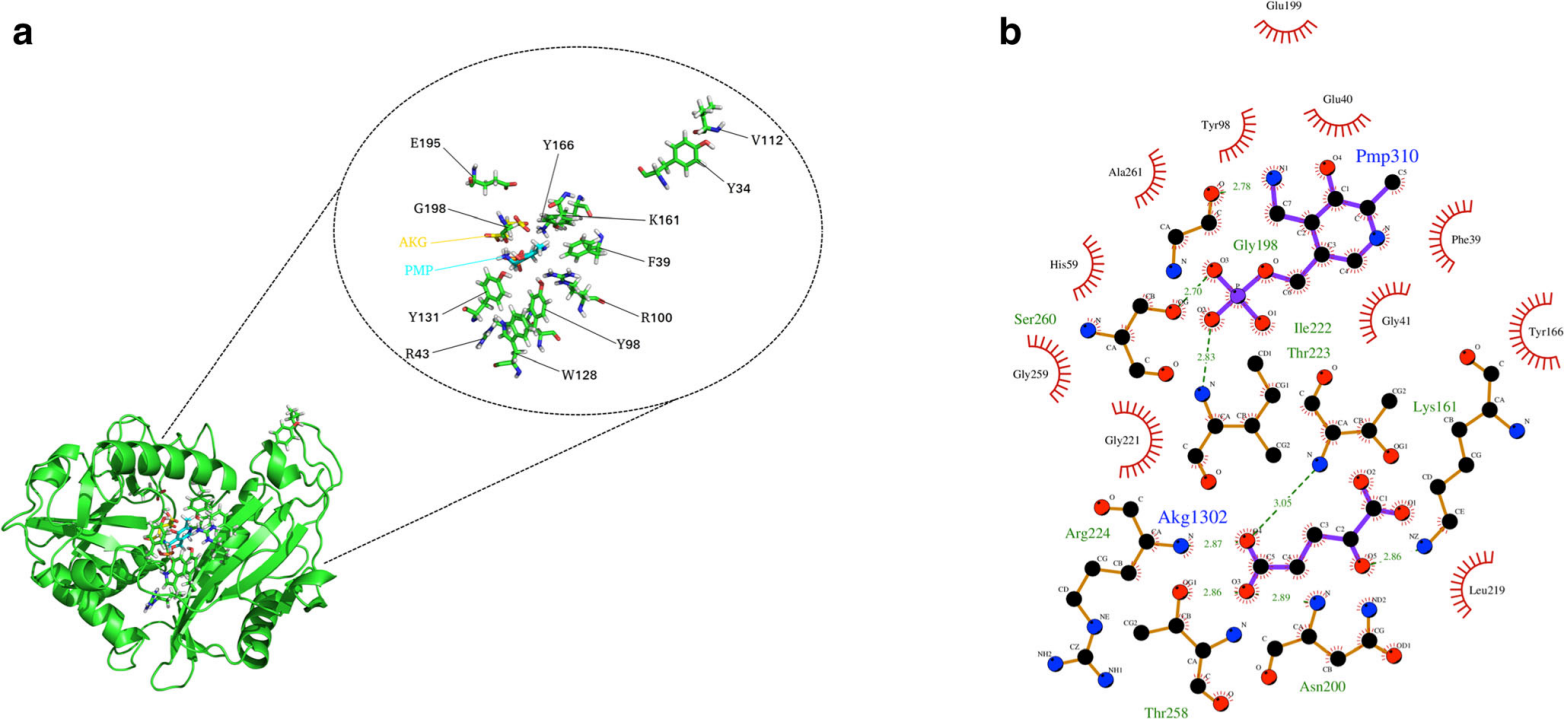
**a** Molecular model of BC61-TAm constructed using I-TASSER server followed by energy minimization using Yasara, showing docked α-KG substrate (AKG) and cofactor (PMP). Models and docked complexes were viewed and manipulated using PyMol. **b** 2D ligand map showing docking and interacting residues between α-KG substrate (Akg1302), cofactor PMP (Pmp310) and BC61-TAm. Construction of residue interaction maps was carried out using LigPlus with subsequent visualisation in PyMol

The primary template utilised for the threading process of the BC61-TAm model was the PDB entry 6NST. Ramachandran analysis of the finalised BC61-TAm model revealed that 95.1% of residues lie within favoured regions, with none in disallowed space. Iterative docking of PMP and α-KG resulted in the production of a predicted final complex (Fig. 4a). LigPlus analysis of the bound complex revealed a number of residue interactions, including contributions by K161, which is a likely candidate for catalysis in the transamination reaction (Fig. 4b).

**Fig. 5:**
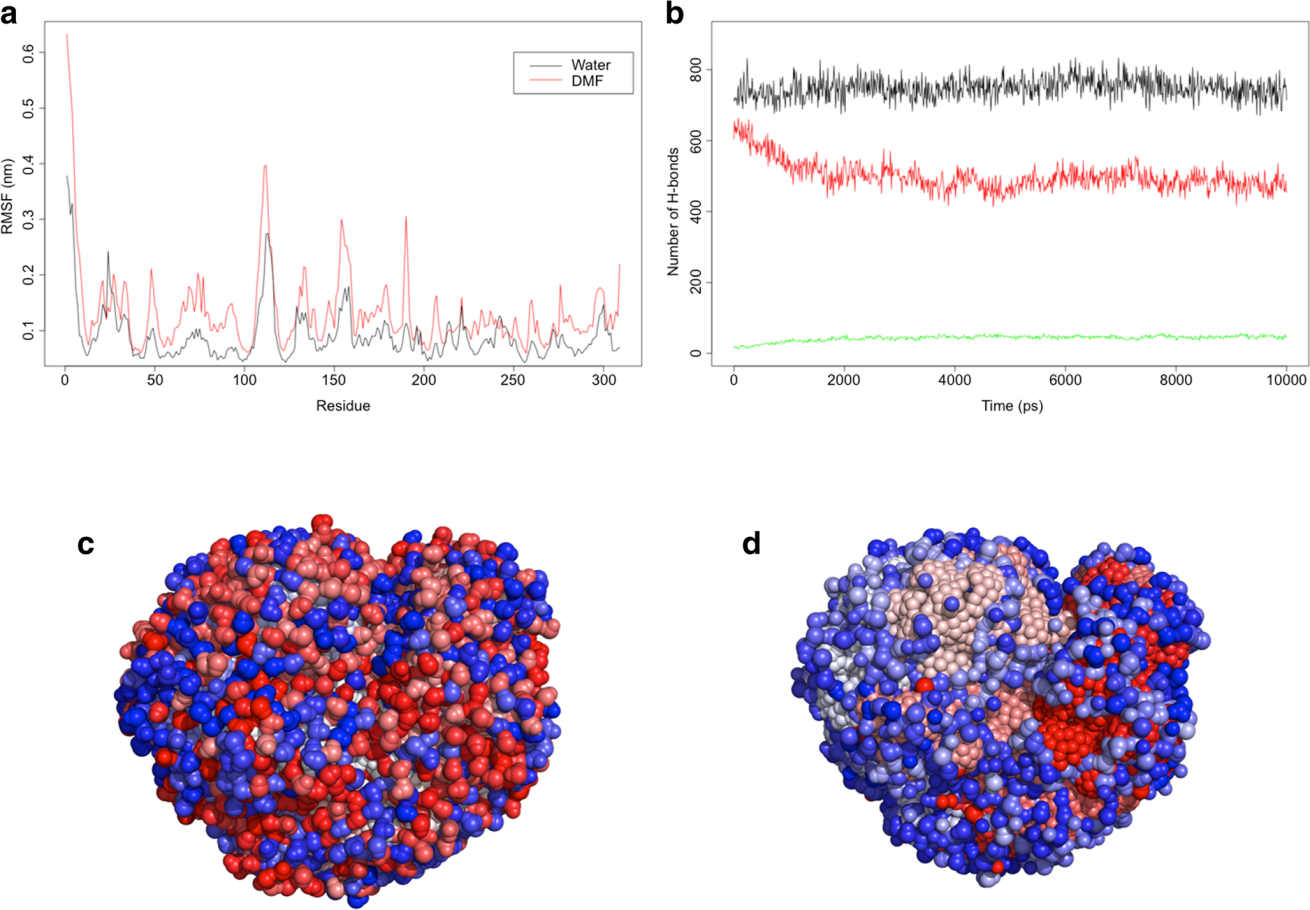
**a** Simulation showing Root Mean Square Fluctuation across individual residues of BC61-TAm in both water and 30% DMF. **b** Simulation predicting number of hydrogen bonds formed between water molecules and BC61-TAm protein in water (black line) and 30% DMF (red line). Number of H bonds between DMF and BC61-TAm in 30% DMF medium is also shown (green line). Solvation shell models simulating BC61-TAm in water (**c**) and 30% DMF (**d**). Water molecules were coloured according to a blue/white/red scheme corresponding to increasing density

In an attempt to elucidate potential explanations for solvent enhanced activity, the BC61-TAm model was subject to molecular dynamics simulations in both water and 30% DMF. Analysis of Replicate Root Mean Square Deviation (RMSD) simulations shows substantial similarity between the water and DMF systems, suggesting they follow similar movement trajectories (Online Resource, Fig. S1). Analysis of average Root Mean Square Fluctuation (RMSF) for the simulations reveals a similar flexibility profile is conserved across all residues in the water and DMF simulations, suggesting similar movement profiles. However, in the DMF simulations, there is increased flexibility compared with that of water alone (Fig. 5a).

The solvation shell surrounding BC61-TAm was simulated in both water and 30% DMF systems, and water molecules were coloured according to a blue/white/red scheme corresponding to increasing density (Fig. 5c, d). In the water only simulation, a universal distribution of water molecules around the protein is observed. Disruption of the solvation shell in comparison with the water-only simulation is apparent, but by far the highest level of water density is observed in the active site cavity. The DMF also appears to contribute to this solvation shell (Online Resource - Fig. S2). The number of hydrogen bonds over time is also calculated for these simulations. When the lower number of water molecules in the DMF simulations is accounted for, the total number of bonds is not significantly different between the two (Fig. 5b). Additionally, analysis of the solvent accessible surface area (SASA) reveals that the DMF simulations possess a higher total SASA than the water-only simulations (Online Resource - Fig. S3).

## Discussion

Of the 19 results obtained using the search term ‘aminotransferase’, those belonging to PLP fold types I and IV were selected for further study, as they are known to be industrially relevant (Steffen-Munsberg et al. 2015). The gene coding for BC61-TAm was annotated as a ‘branched-chain amino acid aminotransferase’ (BCAT). Following cloning and expression, this produced an active protein and was characterised further. As expected, phylogenetic analysis grouped BC61-TAm with PLP fold type IV proteins, and was most closely related to the BCATs (Fig. 2). Residue analysis revealed a considerably higher proportion of the acidic amino acids, aspartate and glutamate, contained within BC61-TAm versus those from the mesophilic bacterial TAms. A greater number of acidic residues on the protein surface is characteristic of haloarchaeal enzymes, with the additional carboxyl group allowing a hydration shell to be stabilised in highly ionic surroundings, thereby maintaining the protein’s solubility at high salinity (Madern et al. 1995; Madigan and Oren 1999; Hough and Danson 1999). Conversely, numbers of basic lysine residues are decreased at the protein’s surface (Danson and Hough 1997; DasSarma and DasSarma 2015). This was also proven to be the case with BC61-TAm when compared with mesophilic TAms.

Although annotated as a BCAT, which utilise branched-chain amino acids as amino donors, BC61-TAm also accepted MBA as amino donor. The ability of BCATs to accept amines as amino group donors has been reported previously in bacterial TAms (Kim et al. 2018), but such a phenomenon has not been described in the archaea. TAms possessing both BCAT and ω-TAm activity can be attributed to the common ancestry shared among PLP fold type IV enzymes (Pavkov-Keller et al. 2016; Kim et al. 2018). Such a relationship is exemplified by the (*R*)-selective ω-TAms, Arth-TAm and At-TAm, and the BCATs, Ec-TAm, Hp-TAm, and BC61-TAm, shown in Fig. 2.

BC61-TAm was shown to be an (*R*)-selective TAm with its ability to utilise (*R*)-MBA as an amino donor, whilst not accepting the (*S*)-isomer. This is a key characteristic of BC61-TAm, given the relative paucity of (*R*)-selective enzymes in the current toolbox and the need for optical purity in many pharmaceuticals.

BC61-TAm displayed a typically halophilic profile when tested over a range of salinities, in that conversion improved with the addition of salt and high relative conversions were still observed up to 4 M NaCl. The solvent tolerance exhibited represents a valuable feature of this enzyme and is characteristic of haloarchaeal enzymes as a whole. This trait also highlights the applicability of BC61-TAm, and similar enzymes, in processes requiring a high concentration of co-solvent, as often seen when substrate solubility is poor.

The usefulness of BC61-TAm is further exemplified by its preference for temperatures beyond the mesophilic range, with optimal conversion seen at 50 **°**C. Halophile-derived enzymes are known to be thermotolerant and, combined with tolerance to organic solvents, possess a profile which could prove extremely advantageous in a number of harsh reaction processes where mesophilic enzymes would fail to function.

Unlike a number of enzymes from halophiles, which also possess an alkaliphilic profile, the optimal pH for BC61-TAm was pH 8.0 (Fig. 3d). This could be explained by BC61-TAm’s thalassohaline source, an environment formed from the evaporation of seawater, whereas alkaliphilic halophiles often derive from soda lakes and perform optimally at pH 9–10 (Hough and Danson 1999).

BC61-TAm was investigated for its ability to aminate a range of diverse aldehyde and ketone substrates, but of the compounds tested, it was only active against α-KG. Despite this limited substrate scope, BC61-TAm shows desirable characteristics for industrial applications in its current form, particularly with respect to its tolerance to salt, organic solvent and increasing temperature, as well as being an (*R*)-selective TAm. In this regard, BC61-TAm represents a superb potential scaffold to which rational design can be applied in order to produce robust, novel biocatalysts.

Residues shown in the ligand map (Fig. 4b) reflect those of previously reported bacterial BCATs. Structural comparisons and searches revealed equivalent residues for all positions (Goto et al. 2003; De Chen et al. 2012), suggesting BC61-TAm likely mediates catalysis according to a typical bacterial TAm pathway. This also suggests haloarchaeal TAms could provide effective scaffolds for rational design, as beneficial amino acid substitutions already known for bacterial TAms should confer the same advantages on TAms from haloarchaea. It is likely that the catalytic pathway of transamination has been conserved in BC61-TAm and has been coupled with the ability to not only tolerate solvents, but be enhanced by the presence of some organic solvents.

Molecular dynamics simulations revealed a number of insights into the improved performance of BC61-TAm in the presence of organic solvent. RMSF simulations showed increased flexibility across individual residues in the 30% DMF simulation compared to water alone. Organic solvents often confer a rigidifying effect on mesophilic proteins, but the increased flexibility observed with BC61-TAm may partially account for the improvement in conversion observed in the presence of DMF.

The hydration shell of a protein is critical to its function, with solvent-mediated disruption of this shell somewhat accounting for the typical rigidity and loss of function associated with most proteins. It seems that in the DMF simulations, BC61-TAm is able to retain a high density of water molecules within the active site cavity, a likely factor in the preservation of its activity despite the presence of solvent. This, along with DMF-mediated interactions, appears to provide a modified water solvent shell that imparts greater flexibility on BC61-TAm. Furthermore, the higher total SASA in the DMF simulation appears to be due to an increased hydrophobic contribution, likely attributable to the DMF. Along with the likely increase in substrate solubility provided by DMF, this provides a feasible mechanistic explanation as to the higher experimental activity observed.

To our knowledge, BC61-TAm is the first haloarchaeal TAm to be studied and characterised for biocatalytic application. BC61-TAm is (*R*)-selective and displays a number of adaptations which could prove useful in industrial processes, including thermotolerance, halophilicity and tolerance to organic solvents. Molecular dynamics simulations have predicted a number of mechanisms by which BC61-TAm exhibits solvent tolerance, a characteristic of particular interest in pharmaceutical biocatalysis. We therefore propose that BC61-TAm can serve as a useful first example of an organic solvent-tolerant, halophilic (*R*)-selective archaeal TAm, whose characteristics can help provide a scaffold for future biocatalyst mining and protein engineering.

## Electronic supplementary material

ESM 1 (PDF 3120 kb)
