# Supplementary material for: Characterisation of a solvent-tolerant haloarchaeal (R)-selective transaminase isolated from a Triassic period salt mine
Source: Appl Microbiol Biotechnol. 2019 May 23;103(14):5727–37. doi: 10.1007/s00253-019-09806-y (PMC6597733; doi:10.1007/s00253-019-09806-y)
Supplement: Supplementary file 1 — (PDF 3120 kb) [file 253_2019_9806_MOESM1_ESM.pdf]

Applied Microbiology and Biotechnology

Electronic Supplementary Material

Characterisation of a solvent tolerant haloarchaeal (*R*)-selective transaminase isolated from a Triassic period salt mine

Stephen A. Kelly,[a] Damian J. Magill,[b] Julianne Megaw,[a] Timofey Skvortsov,[a] Thorsten Allers,[c] John W. McGrath,[b] Christopher C.R. Allen,[b] Thomas. S. Moody,[d/e] and Brendan F. Gilmore [*a]

a. School of Pharmacy, Queen's University Belfast, UK
b. School of Biological Sciences, Queen's University Belfast, UK
c. School of Life Sciences, University of Nottingham, Queen's Medical Centre, Nottingham, UK
d. Almac, Department of Biocatalysis & Isotope Chemistry, 20 Seagoe Industrial Estate, Craigavon, UK
e. Arran Chemical Company Limited, Unit 1 Monksland Industrial Estate, Athlone, Co. Roscommon, Ireland

*Corresponding author
Email: b.gilmore@qub.ac.uk
Tel: 028 9097 2053

Materials and methods supporting information

DNA extratction

Genomic DNA was extracted from CSM-61 using GenElute™ Bacterial Genomic DNA Extraction kit (Sigma-Aldrich, UK) as per manufacturer's instructions. A sample of extracted DNA was sent to MR DNA Lab (Shallowater, TX, USA) for whole genome sequencing (WGS) using the Illumina MiSeq platform and the remainder retained for PCR. The assembled genome was annotated by uploading to the RAST server, and the BC61-TAm gene was identified using the SEED Viewer tool and search term 'aminotransferase'.

PCR amplification and amplicon visualization

2.5 µL (0.5 µM) of each primer along with 2 µL genomic DNA were added to 10 µL 5x Q5 reaction buffer, 1 µL (0.2 mM) dNTPs, 0.5 µL Q5 High-Fidelity Polymerase, 10 µL 5x Q5 high GC enhancer, and nuclease-free water (to 50 µL) (all New England Biolabs Inc., MA, USA). PCR parameters were as follows: initial denaturation at 95 °C for 10 min, 35 cycles of denaturation at 95 °C for 30 s, annealing at 58 °C for 30 s and extension at 72 °C for 1 min 30 s, followed by a final single extension step at 72 °C for 5 min. Amplicons were checked on a 1% agarose gel (90 V, 45 min) and bands of desired length were excised and DNA extracted using a QIAquick Gel Extraction kit (Qiagen, Hilden, Germany) as per manufacturer's instructions.

Ligation of pTA1228 and BC61-TAm and transformation of One Shot® TOP10 Chemically Competent *E. coli* cells

Restricted pTA1228 (1 µL) and restricted BC61-TAm gene (3 µL) were mixed with T4 DNA ligase buffer (1 µL) and T4 DNA ligase (1 µL) (both NEB), and adjusted to 10 µL with ddH_2O followed by overnight incubation at 16 °C. Following incubation, 2 µL of ligation mix was added to 20 µL One Shot® TOP10 Chemically Competent *Escherichia coli* cells and gently pipetted to mix. This was incubated on ice for 30 min, heat shocked at 42 °C for 1 min before returning to ice for a further 2 min. SOC medium was added (200 µL; SOC = 0.5% yeast extract, 2% tryptone, 10 mM NaCl, 2.5 mM KCl, 10 mM $MgCl_2$, 10 mM $MgSO_4$, and 20 mM glucose per litre) and the mixture incubated at 37 °C with rotation for 1 h. The mixture was plated on nutrient agar containing 100 µg/mL ampicillin and incubated overnight at 37 °C. Plasmids were extracted using a QIAprep spin Miniprep kit (Qiagen, Hilden, Germany) following manufacturer's instructions.

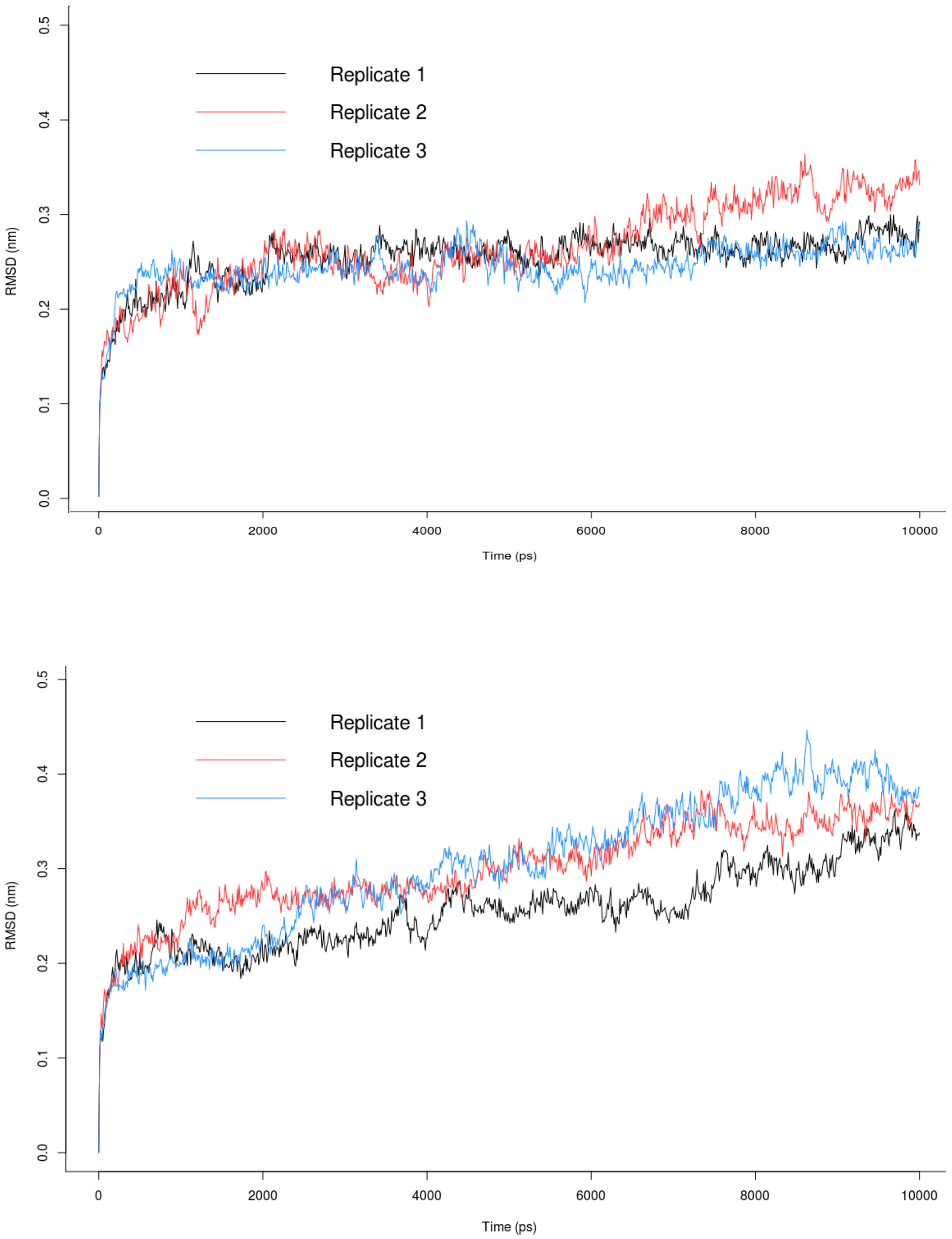

Fig S1. Replicates from Root Mean Square Deviation (RMSD) simulations for BC61-TAm in water (top) and 30% DMF (bottom).

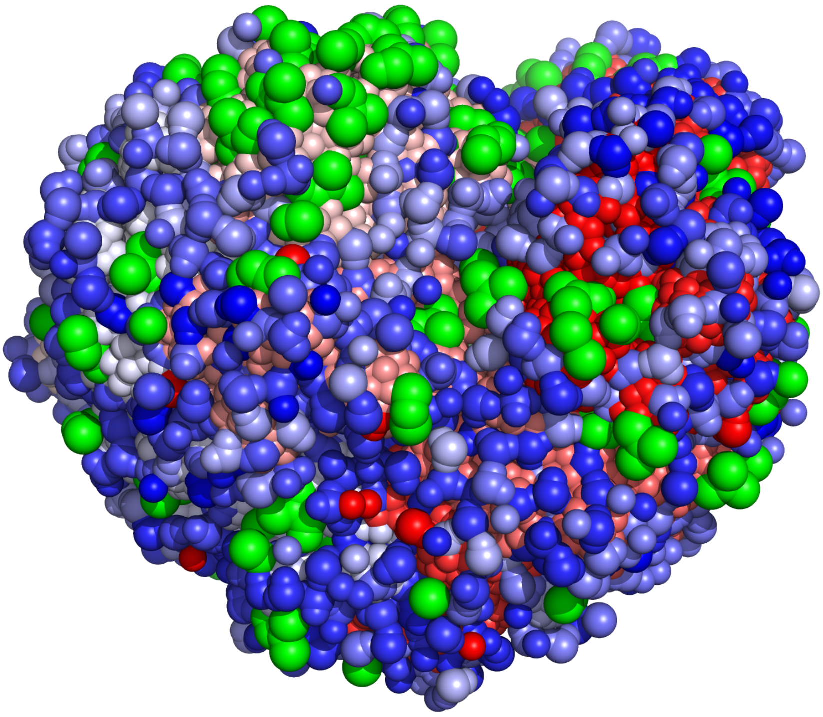

Fig. S2. Solvation shell model simulating BC61-TAm in 30% DMF. Water molecules were coloured according to a blue/white/red scheme corresponding to increasing density, with DMF molecules shown in green.

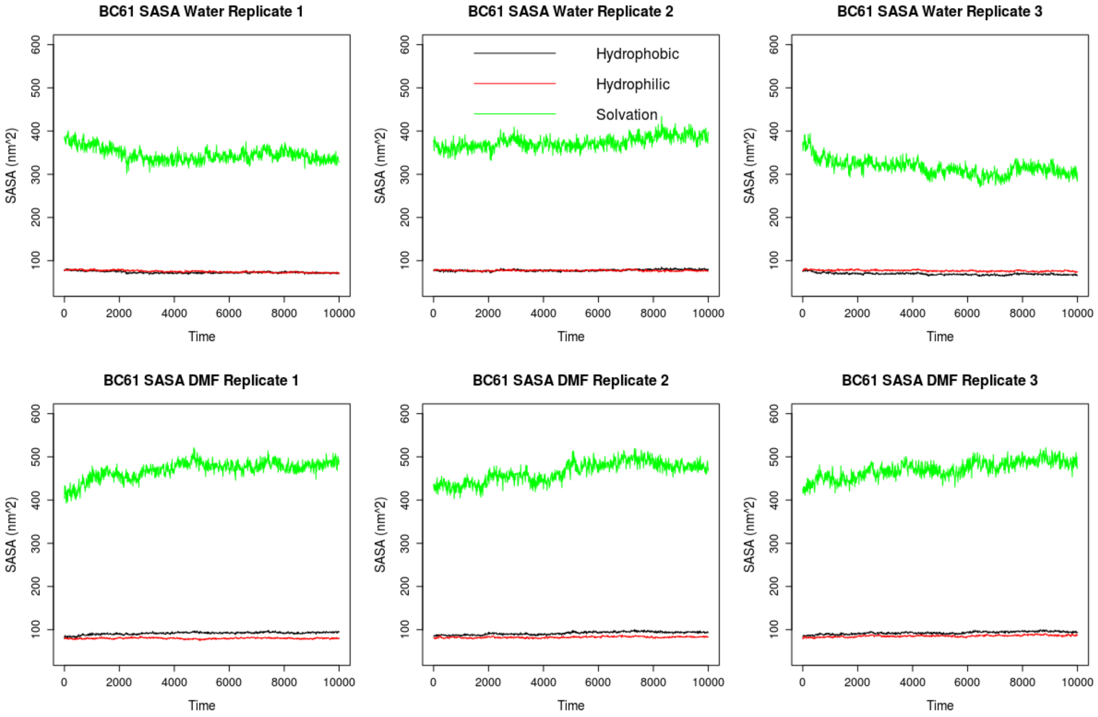

Fig. S3. Replicates of solvent accessible surface area (SASA) simulations for BC61-TAm in both water and 30% DMF.
